# Biomimicry-Based Strategies for Urban Heat Island Mitigation: A Numerical Case Study under Tropical Climate

**DOI:** 10.3390/biomimetics6030048

**Published:** 2021-07-16

**Authors:** Kevin Araque, Paola Palacios, Dafni Mora, Miguel Chen Austin

**Affiliations:** 1 Research Group Energy and Comfort in Bioclimatic Buildings, Faculty of Mechanical Engineering, Universidad Tecnológica de Panamá, Panama City 0819, Panama; kevin.araque@utp.ac.pa (K.A.); paola.palacios@utp.ac.pa (P.P.); dafni.mora@utp.ac.pa (D.M.); 2 Centro de Estudios Multidisciplinarios en Ciencias, Ingeniería y Tecnología (CEMCIT-AIP), Avenida Domingo Díaz, Panama City 0819, Panama

**Keywords:** biomimicry, biomimetics, urban heat island, exterior thermal comfort, equivalent physiological temperature (pet)

## Abstract

In recent years, demographic growth has caused cities to expand their urban areas, increasing the risk of overheating, creating insurmountable microclimatic conditions within the urban area, which is why studies have been carried out on the urban heat island effect (UHI) and its mitigation. Therefore, this research aims to evaluate the cooling potential in the application of strategies based on biomimicry for the microclimate in a historical heritage city of Panama. For this, three case studies (base case, case 1, and case 2) of outdoor thermal comfort were evaluated, in which the Envi-met software was used to emulate and evaluate the thermal performance of these strategies during March (highest temperature month) and October (rainier month). The strategies used were extracted from the contrast of zebra skin, human skin, evaporative cooling, and ant skin. The results showed a reduction of 2.8 °C in the air temperature at 11:00, the radiant temperature decreased by 2.2 °C, and the PET index managed to reduce the thermal comfort indicator among its categories. The importance of thinking based on biomimicry in sustainable strategies is concluded; although significant changes were obtained, high risks of discomfort persist due to the layout and proximity of the building.

## 1. Introduction

In 2018, 55% of the world’s population lived in urban areas, and it is estimated that by 2050 it will increase to 68% [1]. This rapid population increase is the cause of the microclimate in urban regions and will cause an increase in air temperature. Thus, cities and urban areas will be much warmer than nearby rural areas; this phenomenon is known as the urban heat island (UHI) [2]. The UHI can influence people’s health due to thermal stress when there is no environment in buildings and public spaces that provides thermal comfort to their occupants. This increases energy consumption to mitigate high indoor temperatures through air conditioners in buildings.

Panama City does not escape from this situation since it exhibits a particular pattern that concentrates the population in the capital and surrounding areas. In 2010, 65% of the country’s total population lived in urban areas, an action that is expected to increase by 300% by 2050. Historically, the evolution of the capital has been constituted by a vertical development and a closed core of tall buildings with designs sustained by the frequent use of electricity, especially in refrigeration and air conditioning [3].

There are various bioclimatic strategies to mitigate UHI used in urban areas, either by increasing green areas on streets or avenues by studying the effects of vegetation, albedo surface, and orientation of buildings. Such is the case of Tehran city, evaluating different models (the base that represents the current situation, the green cover that covers 50% of vegetation, cold roof model, increasing the roof albedo from 0.3 to 0.6, cold pavement model increasing the albedo for pavement and concrete) and the orientation model comparing the southeast orientation with the southwest [4]. Additionally, considering ecological roofs, cold pavements, and cold roofs that allow sunlight reflection. All these strategies are applicable, but it is essential to emphasize that there is a lack of knowledge related to the changing conceptualization of nature and how this can influence urban planning [5]. 

Like the design based on an understanding of the connection of people with nature called biophilia, GIS (geographic systems information) techniques were used to determine specific areas, sites, and buildings that could be identified as natural sites in the city and geospatial techniques of mapping biophilic cities in Wellington, New Zealand, while a framework of biophilic urbanism was designed allowing the identification of strategic locations facilitating more effective experiences with urban nature and its relevance in other urban contexts [6].

Panama has limited energy resources to meet the growth in demand, and national resources such as wind and solar energy generate important contributions, but only in the long term. Besides, the behavior of the intermittent nature of these energy sources requires considerable support that represents large investments [3]. Evaluations have been performed in Panama considering the bioclimatic of the site for building strategies to assure indoor thermal comfort [7,8], and even to reduce energy consumption due to air conditioning [9], reaching promising results. For this reason, due to scarce resources and because conventional practices are no longer sufficient to solve the problem, it is necessary to change the design thinking and find the most efficient way to solve the problems as nature does, using new strategies based on biomimetics. A recent local study presents a proposal for a multidisciplinary approach and discusses its implementation in Panama [10]. 

Biomimetics can be defined basically as imitation engineering that combines biology and engineering whose goal is sustainability for human development; it should not be confused with the term biomorphism, which is dedicated to the reproduction of organic forms, while biomimetics is responsible for the study of the behavior of nature and how it has evolved over 3.8 billion years.

The inspiration of biomimetic strategies can be represented in the appearance, behavior, shape, or structure of a plant, animal, bacteria, insect, and fungi, as well as mechanisms such as the sweating process-based amygdala. In general, they are referred to as “pinnacles”. Darai Prabhakaran [11] shows how biology can provide bio-inspired ideas in developing materials for buildings, according to the levels of biomimetics. Studies on the properties of materials and how they can influence the building envelope are relevant examples in appearance. Such is the case of the special retro-reflective properties present in flower petals and how they can reflect the heat by radiation in between nearest buildings leading to a reduction of the UHI effect [12]. At the architectural level, in 2019 [13] a study was carried out for a 20-story building in Pakistan, a place characterized by its tropical climate. Generally, glazed buildings offer visual comfort through natural lighting and an excellent view, but they get the solar heat gain. For this, dynamic facades are traditionally created that completely block the sun, but at the same time the visibility to the outside is compromised. The latter is an example of a biomimetic adaptive façade design, with the aim of controlling solar gain without compromising visibility. It is created by means of a module, in which the physical, physiological form and the behavior of the *Oxalis oregana* leaf were imitated. This plant has the ability to track the intensity of sunlight through photoreceptors, its leaf can change from a vertical position (when there is excess light) to a horizontal position (lack of light) in just six minutes, with the objective of enabling more photosynthesis. These qualities were applied to the shading device in such a way that it could change its angles relative to those of the sun.

For the results, the calculations were made by separating the building into three zones in Revit, obtaining the following results: For zone A: it consumes 1,407,779 kWh of electricity per year, of which 60% is from the HVAC; after the update a 27% reduction in HVAC consumption is recorded. For zone B: there is a consumption of 1,471,818 kWh of electricity per year, where 62% belongs to the air conditioning system, achieving a reduction of 32%. For the last zone C: it consumes 1,454,209 kWh of electricity per year, in it the energy consumption by air conditioning is reduced by 29%. In addition to the energy reduction, it was shown that 50% of the floor plan is in visual comfort with an appropriate range of 500–700 lux. This model can be applied to any type of glass building in a hot and humid climate in order to increase its energy efficiency and even combine them with photovoltaic modules integrated into the façade for self-generation. 

Another bio-inspired alternative is passive cooling, where Ali Chesmehzangi and Ayotunde Dawodu [14] carried out a SWOT analysis on how passive cooling through a series of indicators such as health and energy, among others, can have its advantages and disadvantages for urban planning at the macro, meso and micro level.

After exposing the above, this research focuses on mitigating the urban heat island effect and improving the comfort of public spaces (outdoor comfort) based on urban-scale strategies inspired by nature. This inspiration is based on the evaluation of different types of mechanisms or processes through which organisms manage to regulate their temperature. The proposed strategies are evaluated through dynamic simulation for the critical summer month (March) and the critical rain month (October) using Envi-met software in a case study in a historic urban development on the coast of Panama in an approximate area of 66,896 m^2^. 

## 2. Materials and Methods

**Figure 1:**
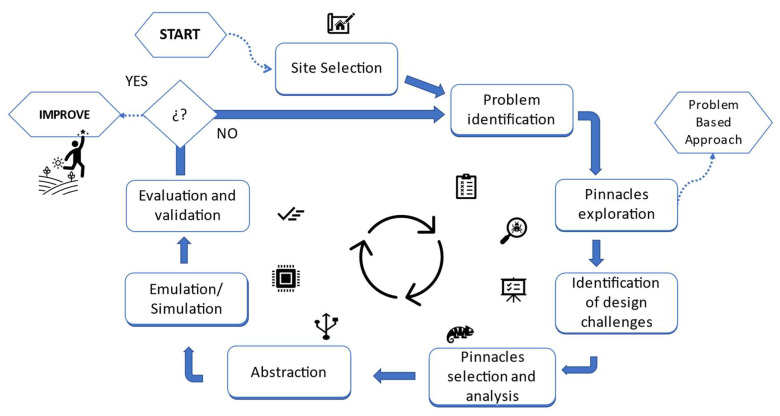
Proposed methodology for the development of the research.

For this research, the methodology adopted is based on the problem-based approach (Figure 1), built upon previous work. It will be developed in the following sections. 

### 2.1. Case Study

The case study considers the historical urban development in Panama City named Casco Antiguo (coordinates 8°57′09″ N 79°32′06″ W). The construction of the Casco was developed under Spanish Crown building regulation in the 16th century, in the mainland territories (Laws of India) created to organize newly conquered lands from an urban point of view [15].

**Figure 2:**
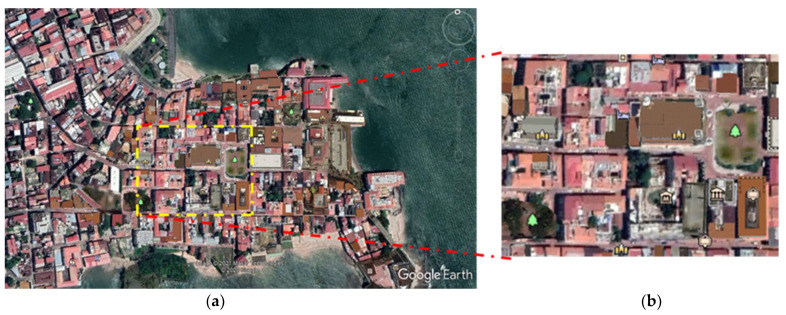
(**a**) Satellite view of the Casco Antiguo; (**b**) Studio cutout view (290 × 226 m).

Currently, the Casco Antiguo is considered a World Heritage Site by UNESCO and is protected by the regulations for preserving historical heritage as a National Monumental Complex. Within the Casco Antiguo, the buildings vary between three and four stories, and the narrow streets of at least 4.5 m to 9 m stand out. The thermal behavior of the urban development within the Casco Antiguo is studied through a section-frame of 290 m (*x* axis) by 226 m (*y* axis), as shown in Figure 2. This section-frame encompasses the Metropolitan Cathedral and the Main “Square Mayor” surroundings, located near the shores of Panama Bay. This area is classified as a climatic zone 3 (LCZ 3) according to Stewart and Oke, due to its type of construction [16], which is described as a dense area of low rise buildings of one to three stories with few trees and primarily covered by pavement and building materials such as concrete, brick, tiles, stone, and cobblestone. In addition, building types are primarily for residential and commercial use.

**Figure 3:**
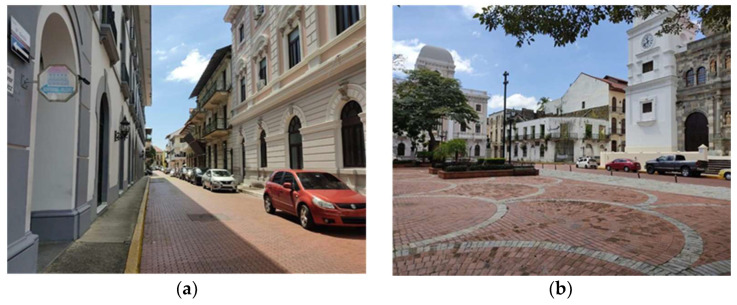
View of the Casco Antiguo: (**a**) streets and (**b**) Square Catedral. Own elaboration.

Regarding the materials of the buildings, most of their roofs are composed of colonial tiles, slabs, wood, although some are zinc with a red antioxidant coating for roofs. The material of the facades varies from calicanto, concrete, clay blocks (Figure 3). The sidewalks are made of concrete, and the streets are mostly red cobblestones and some sections of basalt cobblestones. Table 1 shows materials properties for the case study, which will be used in the simulation (referred here as the base case).

Moreover, thermal comfort indicators were employed to evaluate the proposed strategies’ influence on the exterior comfort, such as air temperature, mean radiant temperature, Tmr, and the comfort index PET (equivalent physiological temperature). For this, the hottest month of the year (March) and the rainiest month (October) were considered and evaluated at the critical hours of 11:00, 15:00, and 16:00. Finally, the study was carried out in the Envi-met microclimate software.

The case study (or base case) was evaluated first through simulation to determine the baseline for each of the comfort indicators employed. Results obtained showed that pedestrians are not in comfort due to the lack of green areas, the existence of urban canyons, building materials for facades and streets. Therefore, this problem will be considered for identifying and searching for solutions that allow mitigating this problem.

### 2.2. Identification and Selection of Biological Analogies 

**Figure 4:**
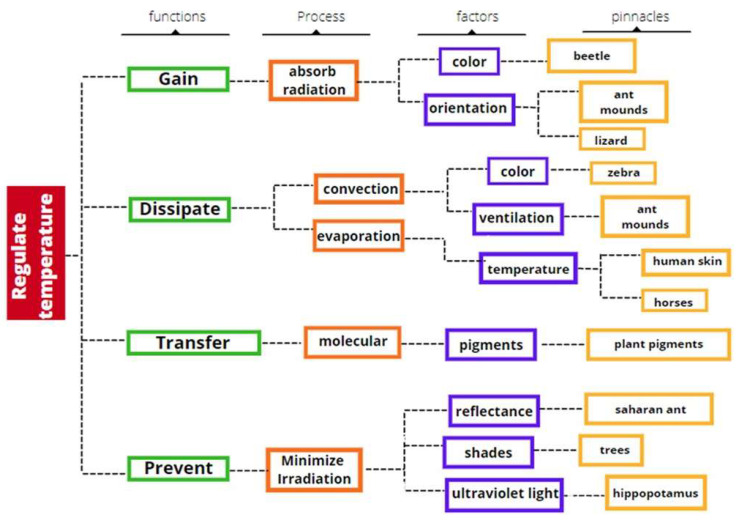
Scanning model for the temperature regulation. Own elaboration.

The identification process begins with the exploration model considering heat regulation as a primary problem as the methodology proposed in [18]. Thus, the biomimetic design is based on four initial functions: gain, dissipate, transfer, and prevent (Figure 4). The exploration model for heat regulation is structured in four levels. The first level describes the functions (for example, heat gain). The second level highlights how each of the functions is carried out (for example, absorbing radiation). The factors exhibited by the highlighted processes are at the third level (for example, color). The fourth level represents the pinnacle or biological analogy with a particular function, such as the beetle to gain heat.

**Figure 5:**
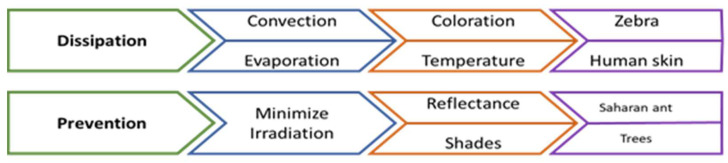
Selected exploration model paths for each design challenge: dissipation and prevention. Own elaboration.

The design challenges chosen are based on preventing solar heat gains in the area studied by reducing the irradiation and increasing the heat dissipation to avoid accumulation in the buildings and streets (Figure 5).

“Prevent heat gain” to avoid heating are characteristic of some species that can be accomplished either through their morphology, physiology, or behavior. The selected process is to minimize solar radiation, in which reflectance and shadows are important factors. Thus, for this design challenge, the selected representative pinnacles are the Saharan ant (reflectance) and the trees (shadows).

The ant is considered the pinnacle that best fulfills the reflectance process and presents values among the highest reflectivities. In our exploratory diagram, the trees minimize solar radiation by increasing the shadow area and are an applicable pinnacle in software for simulation.

“Increase heat dissipation” to remove excessed heat from surfaces is a common characteristic of species called thermoregulation, which can be performed either by morphology, physiology, or behavior. Thus, for this design challenge, the selected processes are: (1) improve convection due to coloration in the zebra, which is the selected pinnacle, and (2) enhance evaporation to cool, just as human skin does through sweating.

Table 2 represents a summary of the selected pinnacles that serve as a quick guide to knowing the principle of each species and its main characteristics, and its objectives in studying design challenges. In which he demonstrates how the zebra and human skin represent the principles and mechanisms of heat dissipation and the ant of the Sahara and the trees the prevention of irradiation.

**Figure 6:**
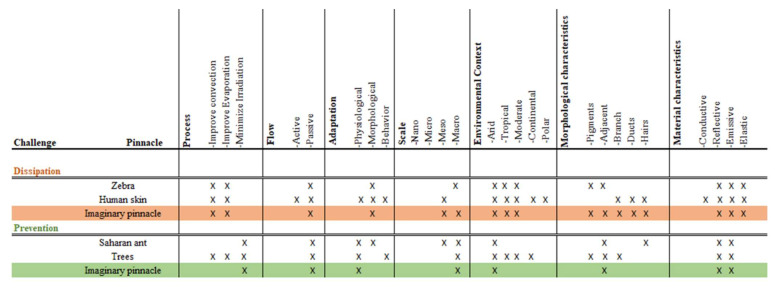
Pinnacles analysis matrix. Own elaboration.

Furthermore, Figure 6 shows the imaginary pinnacles marked in orange and green for the two challenges, dissipation and prevention, respectively. The concept of an imaginary pinnacle is introduced to represent a pinnacle that possesses all the dominant characteristics of both pinnacles chosen for each design challenge in all seven categories (process, flow, adaptation, scale, environmental context, morphological characteristics, and materials characteristics). The most relevant aspects of each pinnacle at each category are marked with an “X”; the imaginary pinnacle will then inherit the superposed aspects of both pinnacles. For each category, the dominant characteristics determine the aspects to be considered: implemented process (e.g., improve convection), flow strategy (passive or active), type of adaptation (e.g., physiological), performance scale (e.g., macro), environmental context (e.g., tropical), morphological characteristics (e.g., adjacent), and material characteristics (e.g., elastic).

Figure 6 corresponds to the design path matrix, which corresponds to the connections of the two imaginary pinnacles for the heat dissipation and prevention challenges. This matrix has the purpose of finding the dominant characteristics to realize the biomimetic design concept. The blue-dotted circles marked the features that have the best agreement among both imaginary pinnacles. The greater the agreement between characteristics, the more dominant it becomes.

The design path matrix for challenges shows the following characteristics and properties relevant to the design concept:

- Passive flow for dissipation and prevention of irradiation;
- For both challenges the scale is relevant;
- The arid and tropical environmental context are considered as most important;
- The morphological characteristic adjacent (or grouped) and pigmented have a greater relationship between the challenges;
- In material characteristics, reflectivity and emissivity describe and work best for both challenges;
- Low thermal conductivity.

### 2.3. Proposed Designs and Simulation

**Figure 7:**
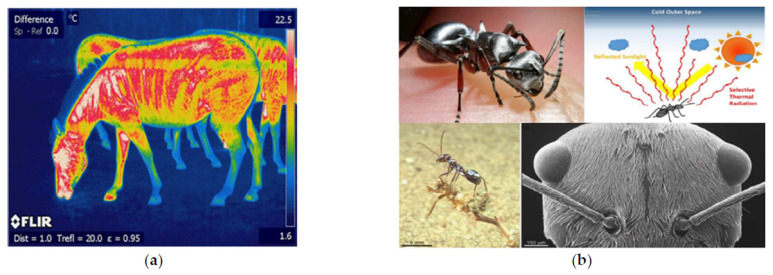
Convective currents due to temperature variation in the black and white lines (**a**). Infrared photo with their respective surface temperature variations (**b**) [18,19,20].

According to the design path matrix (Figure 7), two design cases are proposed. (1) First case (case 1): a coating with high reflectivity and emissivity is applied on the roofs emulating the same properties of the Saharan ant, but with an adjacent or grouping behavior. That is, half of the roof is coated, and the other half remained as the original roof surface. The latter is done interleaved to create a temperature difference, and therefore, the convective currents that will drive the heat evaporation and dissipation as occurring with the zebra (Figure 7). Moreover, trees with dense, rough foliage and light-colored leaves are added to reduce the areas exposed to irradiation and improving transpiration in the area. (2) Second case (case 2): it combines case 1 with a porous pavement with a slight increase in albedo. In this way, a certain amount of incident radiation is reflected, and the evaporation is greater and faster than conventional flooring. Finally, water sources are added, promoting evaporative cooling, just as our body loses heat and regulates temperature.

The meteorological input data for the software Envi-met GmbH (45136 Essen, Germany) [17] were taken from CLIMdata Solargis © service shown in Table 3, the most critical months of the summer season (March) and the rainy season were selected (October) for the dynamic simulation of the base case and the proposed cases. Additionally, the vegetation used in the base model was taken from the Envi-met *Database Manager* that corresponds to Spherical/heart shaped (5 m and 15 m) dense trees with a reflectivity of 0.2 located in both squares; they were also used in both case studies in the addition of more trees.

#### 2.3.1. Case 1 

**Figure 8:**
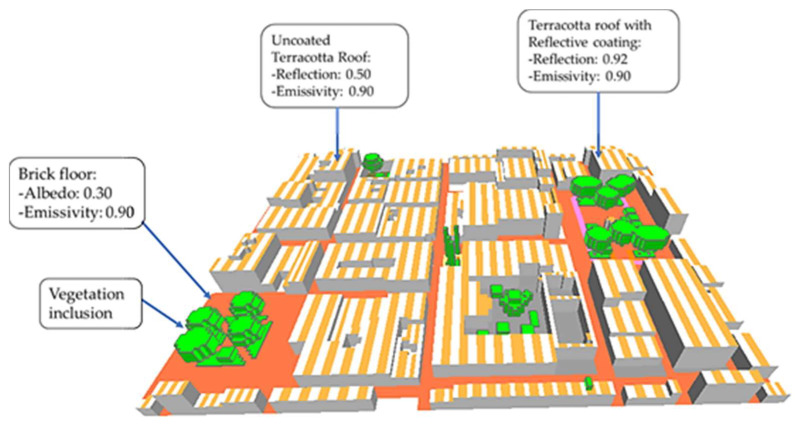
Schematic of case 1 developed in Envi-met.

Modifications were made to the base case, mainly on the roof; a reflective coating replaced the last layer composed of terracotta. Figure 8 shows the outermost layer of the roof with a thickness of 0.01 m, which, together with the other two terracotta layers, has a total thickness of 0.30 m.

The simulation results for the base case evidenced certain zones with a higher temperature than others (presented later in Section 3), and thus, the following is considered: Six trees were placed, two in the Square Cathedral, two in the Square Herrera, and the others in a small space for recreation inside a building. The trees placed in both squares are heart-shaped with a total height of 15 m (trunk and dosel). However, those inside the building are of the same type, but with a height of 5 m, in addition to placing areas with grass in the Square Cathedral and building. The soil remained the same as in the base case.

#### 2.3.2. Case 2

For the construction of case 2, certain characteristics in its design were kept the same as the case 1, such as: the reflective ceiling with the bio-inspired spacing in the zebra stripes, the reflectivity of the Saharan ant, and the shading distribution of trees and lawns. The additional properties included were water fountains both in the cathedral square with two fountains and the Herrera square with a central fountain. The fountains have a water jet height of 4 m (considered by default in the Envi-met library of the Database Manager plugin). Besides, water sprays were added in the critical zones among the streets. 

**Figure 9:**
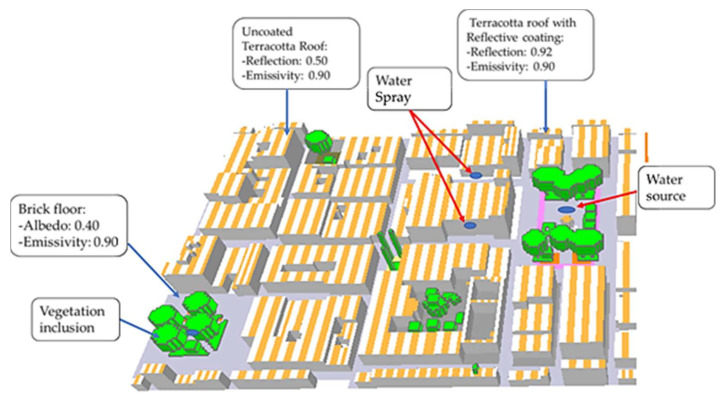
Schematic of case 2 developed in Envi-met.

The spray is at the height of 3 m providing the atomized water for cooling purposes (Figure 9). 

The red brick pavement of the streets (base case) was also replaced by a porous pavement (taken as default from the Envi-met library) to increase evaporative cooling thanks to its water retention. This pavement has an albedo greater than 0.1 with respect to the bricks of the base case (0.3).

**Figure 10:**
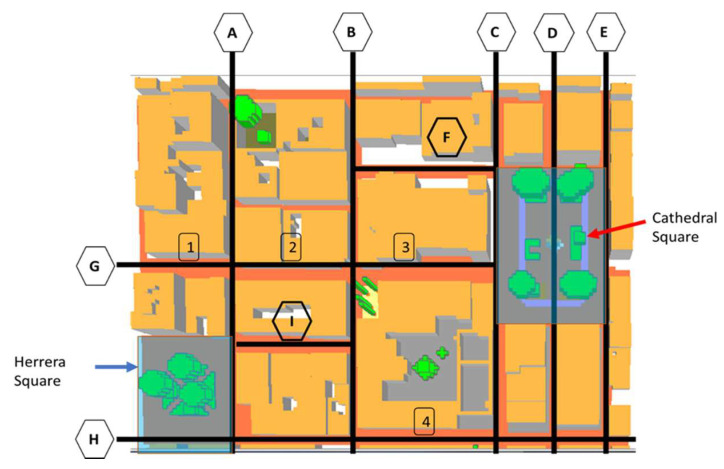
Distribution and arrangement of zones.

To evaluate both the comfort indicators levels and distribution, the results are presented using maps of the section-frame of the Casco Antiguo, organized as shown in Figure 10. For a better interpretation of the results obtained for both cases and to be able to determine a more precise description of the maps shown, in Figure 10, the vertical lines (A,B,C,D,E) represent the streets between the buildings, where the comfort of pedestrians at 1.5 m height in these areas is investigated. The horizontal lines (F,G,H,I) indicate the streets of the section-frame shown, and the numbers (1,2,3,4) represent the blocks. Finally, the areas in blue represent the areas of the squares (Cathedral and Herrera squares) with the same purpose mentioned above.

## 3. Results Analysis and Discussion

This section shows the results obtained for each parameter (air temperature, mean radiative temperature, and PET). The values distribution for each parameter is presented first via maps at 2.5 m, explained using Figure 10. The average value for each map is then compared among each of the cases, presented in tables.

### 3.1. Air Temperature

**Figure 11:**
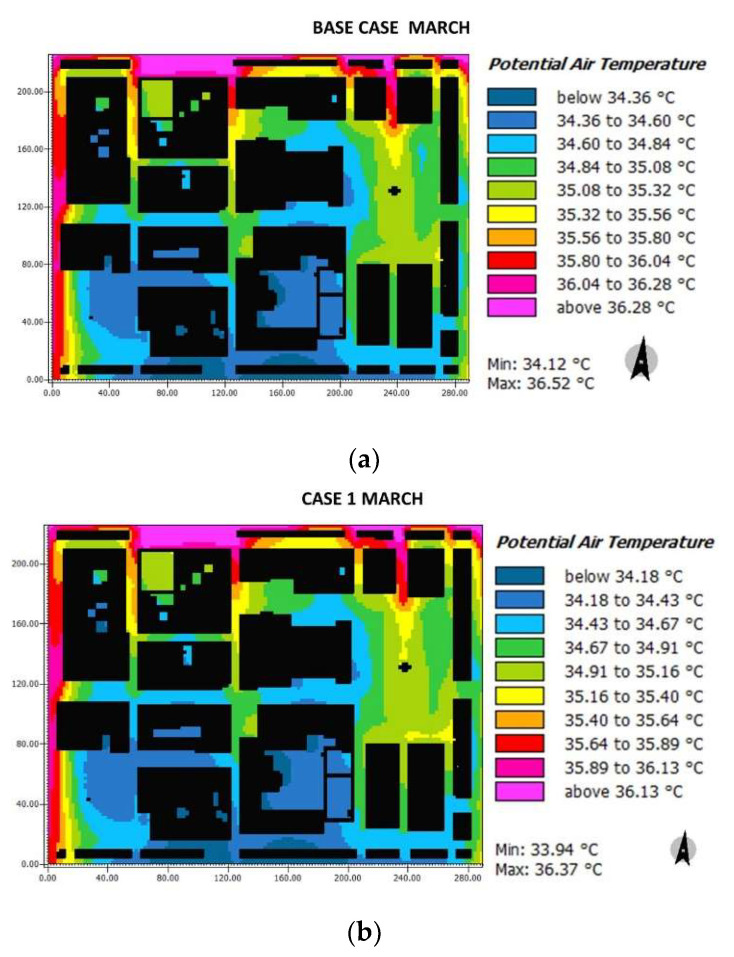

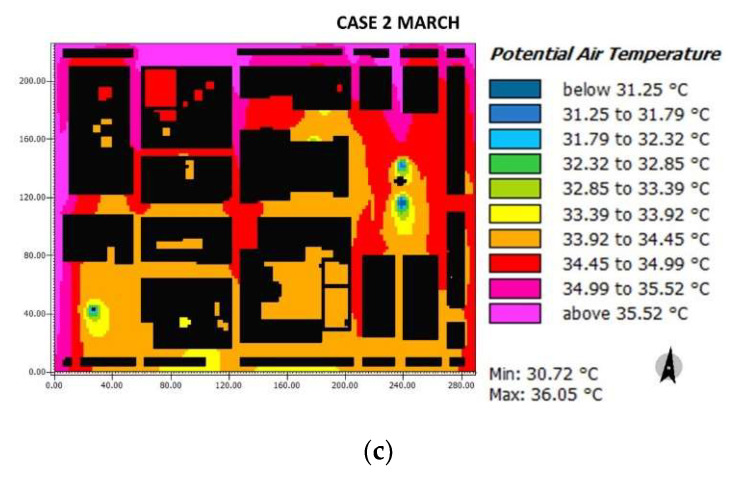
Results maps for potential air temperature for March at 15:00: (**a**) base case, (**b**) case 1, and (**c**) case 2.

Results for the air temperature distribution in each case for March are presented in Figure 11. It can be seen that in the base case, the Herrera square area is around 34.36–35.32 °C, the Cathedral square (34.60–35.56 °C), the Streets G (1, 2 and 3) and F range between 34.36–35.32 °C, streets H and I from 34.36–34.84 °C. In case 1, presented Herrera square values from 34.18–34.91 °C, Cathedral square (34.43–35.40 °C), streets G (1, 2 and 3) and F presented air temperatures between 34.18 °C and 35.16 °C, respectively, and the H, I streets oscillate around 34.18–34.67 °C. In case 2, both squares presented temperatures from 31.25–34.99 °C. It can be seen that the color palette is more uniform due to the change of pavement and the inclusion of water sources, which explains having lower temperature compared to the base case and case 1. Streets G (1–3) and F presented values from 33.92 °C to 34.99 °C, and streets H and I between 33.92 °C and 34.45 °C.

**Figure 12:**
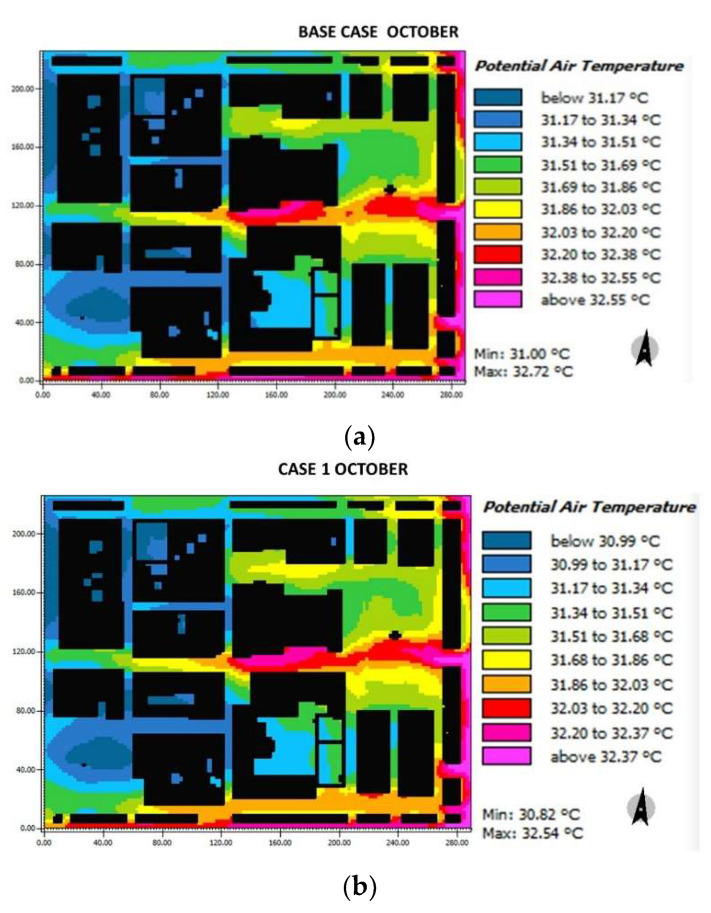

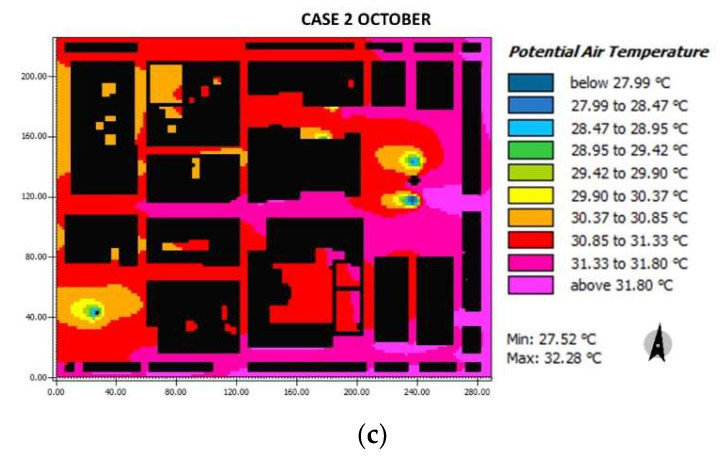
Results maps for potential air temperature for October at 15:00: (**a**) base case, (**b**) case 1, and (**c**) case 2.

In October (Figure 12), for the base case, Herrera square shows a range between 31.17 °C and 31.69 °C, Cathedral square varies between the most critical colors (31.51–32.38 °C), G3 street has a deferential behavior to the others with a range 32.03–32.55 °C, street H oscillates between 31.86 °C and 32.2 °C. Finally, streets A and B (in blue and light blue) show lower ranges between 31.17 °C and 31.51 °C. 

For case 1, Herrera square oscillates between 30.90 °C and 31.34 °C, Cathedral square presents ranges between 31.34 °C and 32.37 °C, the same happens with street G3 with a range of 32.03–32.37 °C, and street H with a range of 31.68–32.03 °C.

Finally, for case 2, a color change in the trim is observed due to the change in the pavement. The squares stand out for their lower range due to the fountains placed, the Herrera square and Cathedral oscillate between 27.99 °C and 31.33 °C and 27.99 °C and 31.8 °C, respectively. While streets G2, G3, H have temperatures of 31.8 °C, street F highlights the added sprays (with a range of 28.95–31.33 °C). For streets A, B the temperature range between 30.37–31.33 °C.

Over the temperature differences shown in Figure 11 and Figure 12 in terms of air temperature, Table 4 summarizes the comparison between each case. A reduction of 1.96 °C in temperature in both squares at 15:00 was possible in case 2 with respect to case base in March and October. This reduction reached up to 2 °C at 16:00 because of the addition of vegetation and water sources. Besides, at 11:00, there was a reduction of 2.69 °C in both squares, and the F street reached a reduction of 1.35 °C. This before demonstrates that significant differences were encountered, thus influencing the biomimicry-based strategies implemented. A temperature difference of at least 0.5 °C can be considered relevant due to human body sensitivity. To the areas that did have high temperatures due to urban canyons, the reflective roof was added, which, although it does not directly influence the height of the pedestrian, represents a contribution to the reduction of the temperature.

### 3.2. Mean Radiant Temperature (Tmr)

**Figure 13:**
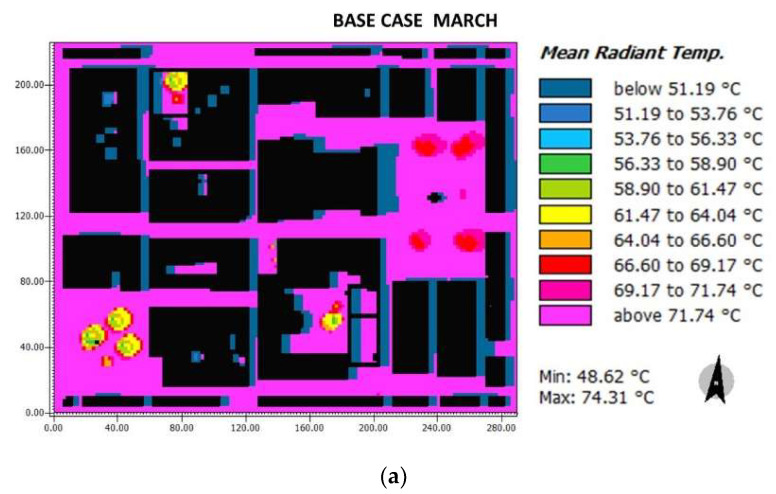

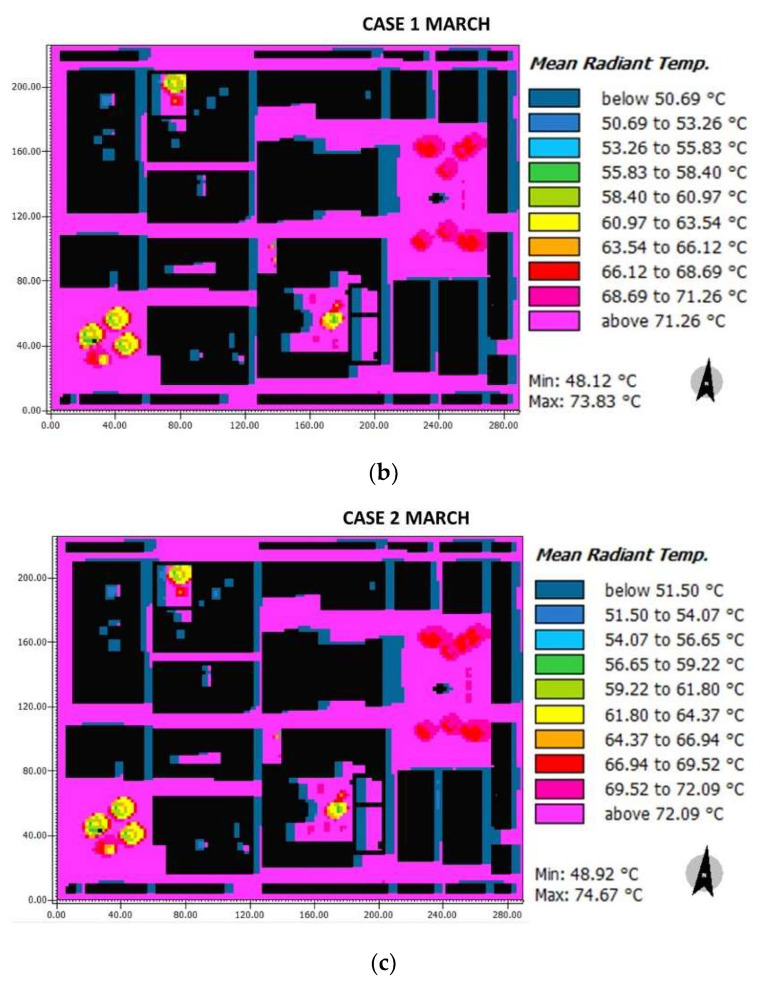
Results maps for radiant mean temperature for March at 15:00: (**a**) base case, (**b**) case 1, and (**c**) case 2.

Regarding the Tmr in March (Figure 13), the three cases did not present substantial differences. The Herrera square (base case: 58.90–71.74 °C; case 1: 58.40–71.26 °C; case 2: 59.22–72.09 °C). The cathedral (base case: 66.6–71.74 °C; case 2: 66.94–72.09 °C), and all horizontal streets G1-G3, H, I (base case: 71.74 °C; case 1: 71.26 °C; case 2: 72.09 °C). On the other hand, the vertical streets (A, B, C, D, and E) presented a blue coloration due to the shadow of the buildings, where the base case: 51.19–53.76 °C; case 1: 50.69–53.26 °C; and case 2: 51.50–54.07 °C. The strategies applied in case 2 seemed to increase the Tmr, however, not significantly.

**Figure 14:**
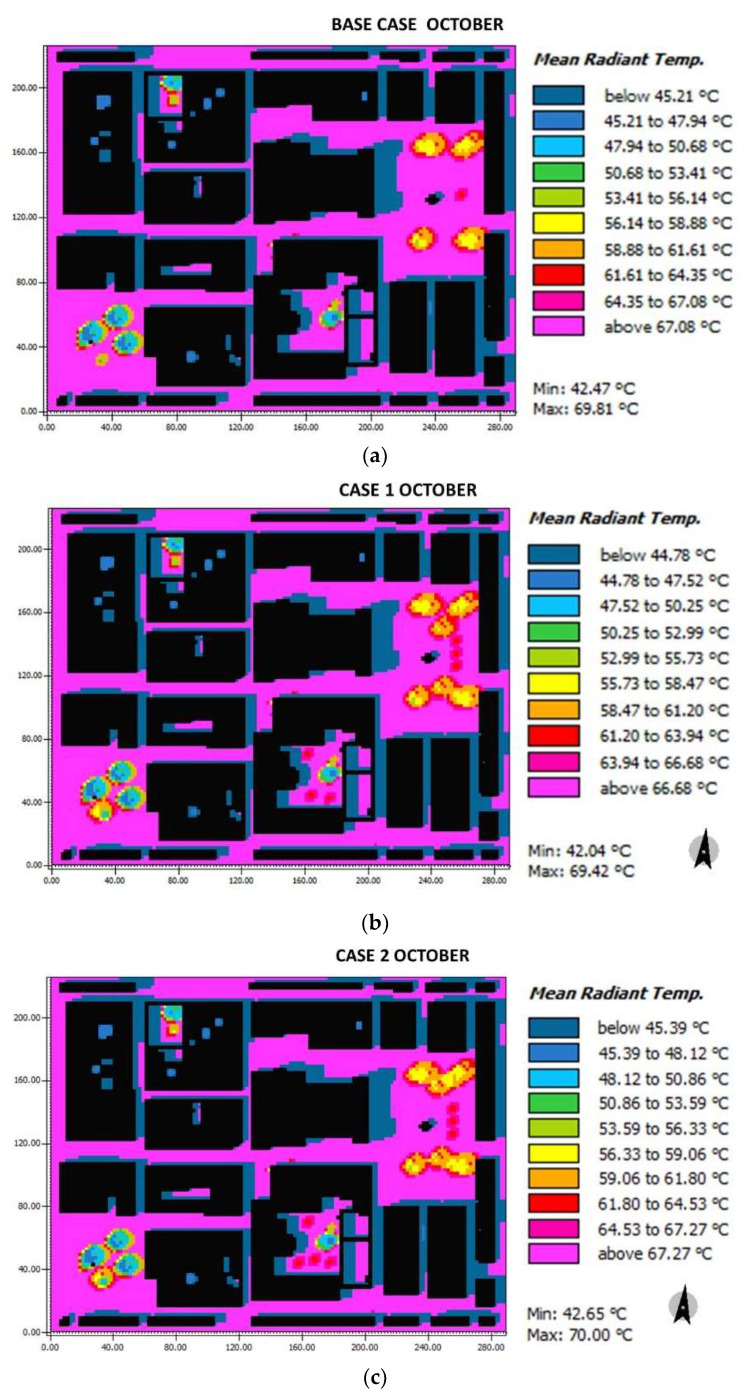
Results maps for mean radiant temperature for October at 15:00: (**a**) base case, (**b**) case 1, and (**c**) case 2.

In October for the base case, the Tmr in the squares due to the presence of trees oscillates between 45.21–64.25 °C for Herrera square and 56.14–67.08 °C for the Cathedral square (Figure 14). For case 1, the Herrera and Cathedral squares vary between 44.78 °C and 63.94 °C and 58.47 °C and 66.68 °C, respectively. For case 2, the changing zones represented by Herrera and Cathedral squares oscillate between 45.39 °C and 64.53 °C and 56.33 °C and 67.27 °C, respectively. In general, the distribution Tmr is around 67 °C.

Table 5 presents the comparison of reduction of the Tmr among the cases. At 11:00, the Tmr reached a reduction of 2.24 °C in March. These Tmr reductions are greater in the morning since the materials are not fully thermally loaded and the high humidity of the hour. 

By 15:00, the change of pavement did not reduce the temperature (in case 2). Actually, the temperature in Square Herrera increased up to 0.6 °C with respect to the base. This increment, although low, is encountered in all the areas and streets. Other alternatives might allow avoiding this before.

Therefore, the latter plays an important role at this hour, when materials are releasing heat to the medium. The Tmr is mainly influenced by the SVF (sky view factor) urban canyon since the buildings are more clustered in the streets around the squares. Moreover, the adjacent vegetation generates shade, and its canopy has greater albedo, allowing less absorption of solar radiation.

### 3.3. Comfort Index PET

For the calculation of the PET thermal comfort index, the BIO-met plugin was used, which is responsible for evaluating and calculating everything related to thermal comfort of people, the skin, and the core generated by the outside environment with the air temperature interior, resulting in the same temperatures. The input data for this plugin were: age of the person (35 years), weight (75 Kg), gender (male), height (1.75 m), surface area (1.91 m^2^), clothing parameters (clo = 0.9) which refers to a person dressed in long-legged underpants, normal shirt, normal pants, socks, shoes, and a light summer jacket as made [25] metabolic rate (164.49 W) of a person walking and met of 1.48, plugging default values.

In calculating this parameter, the simulation results of the air temperature and mean radiant temperature parameters are considered, which in turn depend on the input meteorological data applied for Panama, shown in Table 3, Section 2.3.

**Figure 15:**
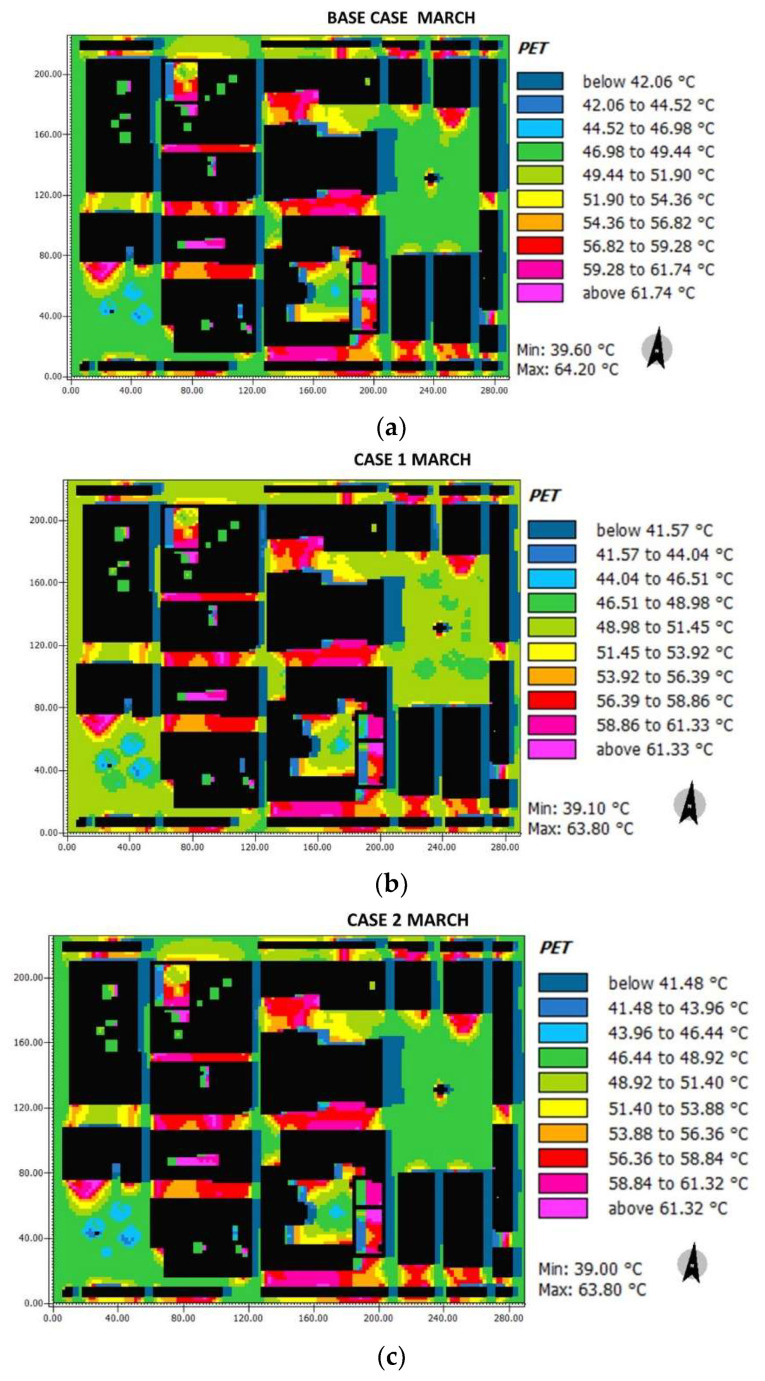
Results maps for pet for March at 15:00: (**a**) base case, (**b**) case 1, and (**c**) case 2.

The resulting values for the PET thermal comfort index in March are presented in Figure 15. Slight PET values fluctuations are encountered in Herrera square (base case: 42.06–51.90 °C; case 1: 41.57–51.45 °C; case 2: 41.48–51.40 °C). Similarly, for Cathedral square, slight decrements in the PET index are found (base case: 46.98–51.90 °C; case 1: 46.51–51.45 °C; case 2: 46.44–48.92 °C). However, significant increments are found for all horizontal streets with respect to both the Herrera and Cathedral squares (base case: 51.90–61.74 °C; case 1: 51.45–61.33 °C; case 2: 51.40–61.32 °C), while the vertical streets A, B, C, D, and E presented no significant changes (base case: 42.06–44.52 °C; case 1: 41.57–44.04 °C; case 2: 41.48–43.96 °C).

**Figure 16:**
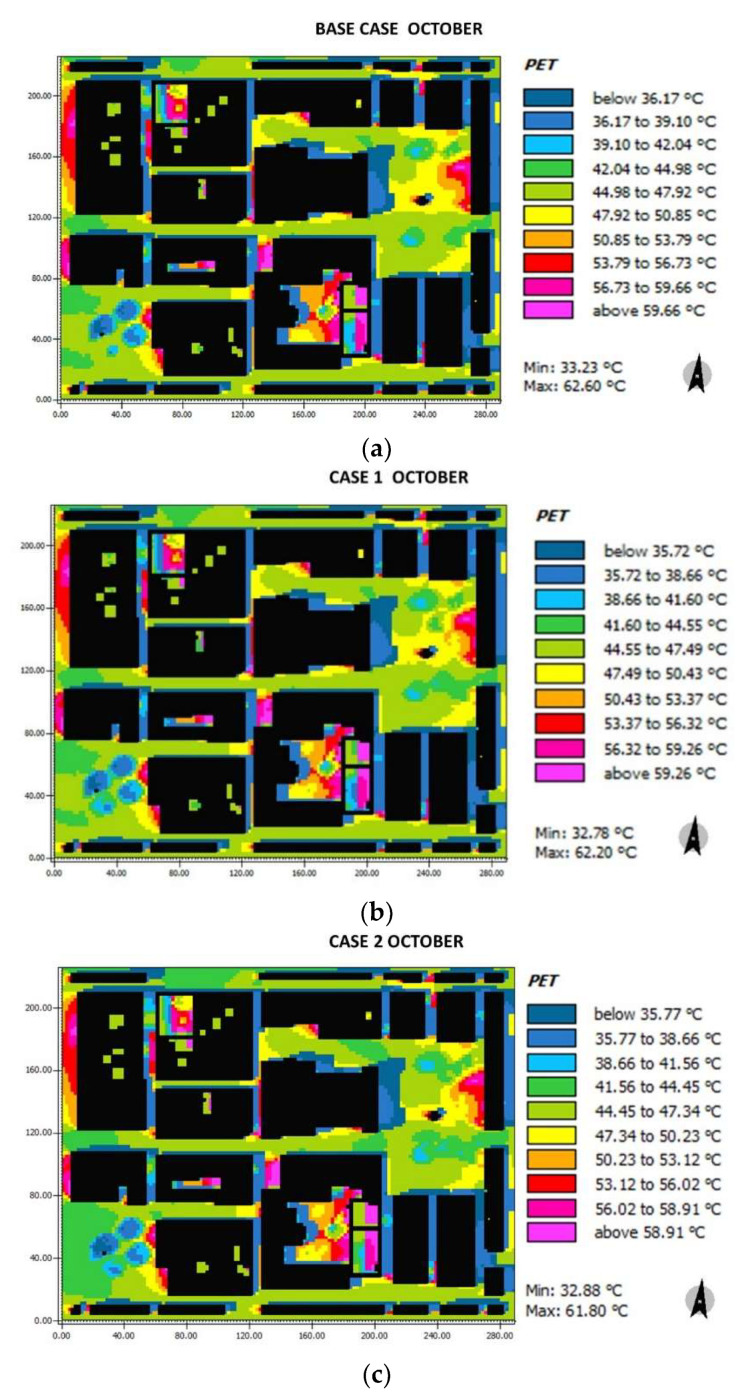
Results maps for pet, for October at 15:00: (**a**)base case, (**b**) case 1, and (**c**) case 2.

For the PET comfort index in October (Figure 16), the base case ranges between 36.17 °C and 47.92 °C and 39.1 °C and 56.73 °C for Herrera and Cathedral squares, respectively. The horizontal streets (H, G1, G2, G3, I) present a range of 44.98–47.92 °C unlike the vertical ones (A, B, C, D, E), in which, due to the generated shadow, lower values are found (36.17–39.1 °C). For case 1, in Herrera square, the PET oscillates between 35.72–50.43 °C, for the Cathedral square (38.66–56.02 °C), and the horizontal streets (H, G1, G2, G3, I) (44.45–47.34 °C) and for the vertical streets (35.77–41.56 °C). Finally, for case 2, in both squares, the PET values oscillate between 35.77–47.34 °C; the streets H, G1, G2, G3, I, the PET varies from 44.45 °C to 47.34 °C, and finally, for the streets, A, B, C, D and E, the PET values present a range of 5.77–38.66 °C.

For the comparison of this indicator, values and ranges were taken from a study in hot and humid regions [26] in which different indices of outdoor comfort and ranges that offer guidelines to choose the most appropriate according to the region are reviewed.

At 15:00, only one of the eight zones presented a reduction in the PET thermal comfort index in October (Table 6). Only Herrera square stood out in reducing the thermal comfort index of PET from extreme heat to high heat, that is, less than 42 °C, thanks to water sources, vegetation, and the change of pavement. However, in March, in Cathedral square, the PET decreased by 1.7 °C with respect to the base case, the comfort indicator could not be reduced, remaining above 42 °C (extreme heat). The same happened in the Cathedral square in March; a considerable drop was found (2 °C), but it did not manage to drop below 42 °C. In addition, for streets A, B, C, D, and E the indicator did decrease in relation to March, with October being moderately hot (below 38 °C) because it was the most humid month.

## 4. Conclusions

This research’s main objective was to conceptualize and evaluate heat mitigation through biomimetic strategies on an urban scale in the Casco Antiguo in Panama City. The biomimicry-based strategies were developed using the problem-based approach. Among the identified problems to be due to the urban canyons, materials of the facades, streets, and absence of vegetation. These features have a direct impact on the exterior comfort in the study area. For this reason, biomimicry appears as an alternative for passive and low consumption solutions based on nature.

From this, two design proposals were presented in two cases as follows (emulation): Vegetation and reflective roofs were added, emulating the behavior of the Saharan zebra and ant (case 1). The second consisted of mixing the first case plus an additional passive strategy that is the porous pavement for evaporative cooling, in addition, an active strategy that consisted of adding spray and fountains (case 2).

With the simulation of each design proposal, the following major results were obtained:

- The Herrera and Cathedral squares, in March for the air temperature, presented a reduction of 2.68 °C compared to the base case;
- The mean radiant temperature (Tmr) increased in 0.6 °C, in March, in Herrera square and, in the rest of the areas, the Tmr had an approximate increase of 0.3 °C, due to the high radiation at 15:00, and also, due to the pavement applied in case 2. An albedo 0.1 greater (case 2) than in the base case, served to reduce the Tmr in 2 °C by 11:00 and almost 1 °C by 16:00;
- Regarding the indicator PET (equivalent physiological temperature), in October, six zones (squares and streets) presented a change from extreme to high heat and from high to moderate heat. However, in March, for case 2, the strategies implemented only managed to lower one indicator, due to the high temperatures.

It should be noted that the applied strategies considered individually (for example, porous pavement) had lower but significant results, and overall better results were obtained concerning the base case.

Biomimetics is important since nature, throughout its history, has been adapting and solving its problems passively and without negative impacts on itself. This is also the case because through its methodology, any application area (architecture, engineering, biology) can use it based on its approaches based on problems or solutions to provide energy-efficient and sustainable designs.

It is important to note that the data obtained are the product of a simulation and no type of comparison is made with another similar study using the software, only PET ranges similar to a study are taken for a region with a hot and humid climate.

Finally, although considerable reductions in the heat and temperature stress indexes were achieved, high levels of discomfort persist, so it is recommended for future research to use the design methodology based on the same or other biomimetic strategies in the construction of enclosures (walls) to consider cloudiness and rain in the software, and obtain experimental data through meteorological measurements in order to obtain more precise results. This would be done in addition to the evaluation of the behavior of walls and green roofs versus reflective materials in this case study. Furthermore, future work should consider evaluating how the use of these strategies affects energy efficiency in buildings and the reduction of electricity consumption by air conditioners. 

## Figures and Tables

**Table 1:** Materials for the current case/cutout base.

Elements	Materials	Envi-Met Software Library Data [17]	Emissivity	Reflectivity
Pavements	Red cobblestones	Brick road (red stones)	0.9	0.3
	Concrete floor	Concrete pavement gray	0.9	0.9
	Basalt floor	Basalt brick road	-	0.8
Roof	Terracotta	Roofing: Terracota	0.9	0.5
Vegetation	Low leafy trees	Spherical/Heart-Shaped (5 m), dense	-	0.2
	Tall leafy trees	Spherical/Heart-Shaped (15 m), dense	-	0.2
	Clay floor	Loamy soil	-	0.2

**Table 2:** Summary of the Pinnacles Analysis.

Pinnacles Strategy	Mechanism	Fundamental Principles	Main Feature
Zebra	Black and white streaking causes a temperature differential [19,20].	Convective currents are caused by increasing evaporation.	High convection and evaporation
Arrangement of animal stripes for heat regulation			
Human skin	Reaction by stimulation of the hypothalamus and the body’s heat sensors [21,22]	Allows the loss of heat by conduction and evaporation of sweat	High convection and evaporation
Sweating or perspiration for heat regulation			
Saharan ant	High reflection in the NIR range and emissivity in the NIR [23]	Reflection of thermal radiation and high emissivity to release excess heat	High reflectivity and emissivity
Silver hairs with triangular structure			
Trees	Vegetation cover to increase shadows	Reduce the areas of exposure to irradiation, improve transpiration [24]	Shades
Plant properties: foliage density, roughness, leaf clarity, thickness.			

**Table 3:** Monthly meteorological critical values.

Months	Tmax (°C) Hour	Tmin (°C) Hour	HRmax (%) Hour	HRmin (%) Hour	Wind Speed (m/s)	Wind Direction (°)
January	35 15:00	23.9 6:00	94 6:00	44 15:00	5.9	114
February	34.6 15:00	22.2 6:00	93 6:00	40 15:00	5.6	60
March	35.6 15:00	24.9 6:00	73 6:00	36 16:00	5	350
April	35.3 16:00	24.8 6:00	82 24:00	44 16:00	2.8	117
May	34.8 15:00	24.5 6:00	90 6:00	53 16:00	3.9	78
June	32.8 15:00	23.4 6:00	94 6:00	58 15:00	2	92
July	35.5 16:00	24.3 6:00	97 7:00	49 16:00	1.8	116
August	34.3 15:00	24.1 6:00	95 5:00	52 15:00	4.9	118
September	32.5 15:00	23 6:00	98 24:00	60 16:00	3.1	116
October	32.5 15:00	23 6:00	96 6:00	62 14:00	4.4	90
November	32.9 15:00	23.7 6:00	94 5:00	61 13:00	9.2	73
December	34.3 15:00	24.6 6:00	94 7:00	50 16:00	6.7	26

**Table 4:** Comparison of air temperature for base case and case 2 by streets for critical months.

Period of the Day		Area/Street	Reduction (°C)
March	11:00	Herrera Square	2.68
		Cathedral Square	
		A, B, C, D, E	0.87
		F	1.35
		G1–G3, H, I	0.78
	15:00	Herrera Square	1.72
		Cathedral Square	1.96
		G1–G3, F	0.38
		H, I	0.41
	16:00	Herrera Square	2.2
		Cathedral Square	2.2
October	11:00	Herrera Square	1.79
		Cathedral Square	
		G3	1.2
	15:00	Herrera Square	1.7
		Cathedral Square	2
		A, B	0.5
		G3	0.5
		H	0.2
	16:00	Herrera Square	2.03
		Cathedral Square	

**Table 5:** Comparison of the mean radiant temperature for base case and case two by streets for critical months.

Period of the Day		Area/Street	Reduction (−)/Increase (+) (°C)
March	11:00	Squares (Herrera/Cathedral)	−2.24
	15:00	Herrera Square	0.6
		G1–G3, H, I	0.35
		Cathedral Square	0.34
		A, B, C, D, E	0.31
	16:00	Square (Herrera/Cathedral)	−0.97
		A, B, C, D, E	−0.88
October	11:00	Square (Herrera/Cathedral)	−1.66
	15:00	Herrera Square	0.23
		Cathedral Square	0.3
		A, B, C, D, E	0.2
		G1–G3, H, I, F	0.2
	16:00	Square (Herrera/Cathedral)	−0.1

**Table 6:** Comparison for PET for base case and case 2 by streets for critical months.

Month	PET		Thermal Stress		Reduction (°C)	Did the Indicator Decrease?
	Time	Zone	Base Case	Case 2		
March	11:00	Herrera square	High Heat	High Heat	−2.01	NO
		Cathedral square	Extreme	High Heat	−2.14	YES
		G1	Extreme	Extreme	−2.38	NO
		G2, G3, H4, I	Extreme	Extreme	−2.44	NO
	15:00	Herrera square	Extreme	Extreme	0.5	NO
		Cathedral square	Extreme	Extreme	1.7	NO
		G1, G2, G3, H, I	Extreme	Extreme	0.46	NO
		A, B, C, D, E	Extreme	Extreme	0.57	NO
	16:00	Herrera square	Extreme	Extreme	−0.5	NO
		Cathedral square	Extreme	Extreme	−0.5	NO
		G1	Extreme	Extreme	−0.6	NO
		G2, G3, H4, I	Extreme	Extreme	−1.89	NO
		A, B, C, D, E	High Heat	High Heat	−0.39	NO
October	11:00	Herrera square	Moderate Heat	Moderate Heat	−2.39	NO
		Cathedral square	High Heat	Moderate Heat	−1.1	YES
		A	Extreme	Extreme	−4.08	NO
		B, C	Extreme	Extreme	−1.37	NO
		D, E	Extreme	High Heat	−1.22	YES
	15:00	Herrera square	Extreme	High Heat	0.54	YES
		Cathedral square	Extreme	Extreme	2	NO
		G1, G2, G3, H, I	Extreme	Extreme	0.5	NO
		A, B, C, D, E	Moderate Heat	Moderate Heat	0.7	NO
	16:00	Herrera square	High Heat	Moderate Heat	−1.62	YES
		Cathedral square	Extreme	High Heat	−1.99	YES
		A, B, C, D, E	Moderate Heat	Moderate Heat	−0.15	NO
		G1	High Heat	Moderate Heat	−1.61	YES
		G2, G3, H4, I	Extreme	Extreme	−0.31	NO

## Data Availability

Not Applicable.
